# A Bayesian Measure of Model Accuracy

**DOI:** 10.3390/e26060510

**Published:** 2024-06-12

**Authors:** Gabriel Hideki Vatanabe Brunello, Eduardo Yoshio Nakano

**Affiliations:** Department of Statistics, University of Brasília, Campus Darcy Ribeiro, Asa Norte, Brasília 70910-900, Brazil; ghvbrunello@gmail.com

**Keywords:** Bayesian inference, credible interval, goodness of fit, regression models, 62F15

## Abstract

Ensuring that the proposed probabilistic model accurately represents the problem is a critical step in statistical modeling, as choosing a poorly fitting model can have significant repercussions on the decision-making process. The primary objective of statistical modeling often revolves around predicting new observations, highlighting the importance of assessing the model’s accuracy. However, current methods for evaluating predictive ability typically involve model comparison, which may not guarantee a good model selection. This work presents an accuracy measure designed for evaluating a model’s predictive capability. This measure, which is straightforward and easy to understand, includes a decision criterion for model rejection. The development of this proposal adopts a Bayesian perspective of inference, elucidating the underlying concepts and outlining the necessary procedures for application. To illustrate its utility, the proposed methodology was applied to real-world data, facilitating an assessment of its practicality in real-world scenarios.

## 1. Introduction

For effective decision making, a thorough understanding of the problem is crucial. However, this understanding often requires dealing with a significant amount of data, which, due to their volume, present complex relationships. In such scenarios, recognizing crucial data relationships may not be straightforward, necessitating the application of analytical methodologies. Statistical modeling stands as a valuable asset in this context, streamlining complex events through the lens of hypothetical probabilistic models. These models find validation through rigorous empirical observation, enhancing their reliability and utility. Therefore, it is essential to verify that the chosen model adequately represents the problem of interest. Failure to specify the model correctly can compromise the quality of information obtained, leading to inaccuracies, and ultimately, erroneous conclusions. Various methods exist to evaluate the quality of a model, but most involve subjective classification criteria or complex elaboration, deterring their use in practical applications. Hence, this work will introduce a proposal for a Bayesian methodology to evaluate the quality of a statistical model based on its predictive ability. This means assessing the model’s effectiveness in predicting values for new instances of the problem at hand. The advantage of this proposal lies in its simplicity. By focusing on the model’s predictive capacity, it does not solely rely on its fit to existing data. This approach streamlines its application and promotes its use in decision-making scenarios.

The proposal outlined in this work is a modification of an external validation approach proposed by [1], which lacks an objective criterion for assessing the model’s quality. Additionally, this methodology shares a similar logic to the Log Pseudo Marginal Likelihood (LPML) [2], but with distinct objectives. Whereas the LPML compares models, the aim of this work’s proposal is to determine whether a model can accurately predict a new observation. The behavior of the accuracy measure was examined through simulated applications in generalized linear models with exponential distribution.

The goals of this work were to introduce a proposal for a Bayesian methodology for assessing model adequacy based on its predictive ability, investigate the performance of this methodology in generalized linear models with exponential distribution, and devise a straightforward criterion for the methodology to assess the quality of a model. The proposed classification criterion was derived from simulated data and was demonstrated using a real dataset from the literature. All simulations and analyses were conducted using the open-source software R [3].

### Assessment of the Quality of Statistical Models

The assessment of model quality is an area with extensive literature within the frequentist paradigm, with numerous techniques available for objective evaluation. For example, D’Agostino’s book [4] provides an overview of the most important classical goodness-of-fit tests. Conversely, within the Bayesian framework, the literature is relatively recent, and the existing methods are often restrictive or complex, resulting in this fundamental aspect of statistical analysis occasionally being neglected.

In a Bayesian context, evaluating the quality of a model does not rely on the adequacy of the likelihood function used, unlike the classical approach. Instead, it depends on the suitability of the posterior distribution, as any relevant inference for the problem stems from it. Additionally, some authors propose that the quality of a Bayesian model should be judged based on its predictive distribution. If the data do not align with the predictive distribution, it is anticipated that the model is not appropriate [5].

Furthermore, additional methods for evaluating the quality of a model in a Bayesian framework are discussed in [6]. Some of the commonly used techniques include Dirichlet Processes [7], posterior predictive check [1], Log Pseudo Marginal Likelihood [2], leave-one-out (LOO) cross-validation, and the Widely Applicable Information Criterion (WAIC) [8].

Dirichlet Processes [7] are utilized in estimating a non-parametric Bayesian model, which is subsequently compared to the proposed model using the Bayes Factor [9] to assess if their difference is significant. This method serves as a model fitting technique that employs the difference between the values estimated by the proposed model and those by the non-parametric model as a quality criterion. Nonetheless, it is associated with the drawback of necessitating a complex process for its development.

The posterior predictive check [1] assesses whether any T statistic from the model is consistent with the empirically observed data. This method entails a dual use of the data, as they are utilized both in the model estimation process and for comparison with the test statistic. The subjectivity in defining the T statistic is a notable critique of this approach, as it must be adapted to each specific problem.

The leave-one-out (LOO) cross-validation and the Widely Applicable information Criterion (WAIC) [8] are methods that estimate pointwise out-of-sample prediction accuracy. According to Vehtari et al. [10], these methods were less used in practice because they involve additional computational steps. In order to mitigate this problem, they presented an optimized computation method for LOO using Pareto-smoothed importance sampling.

The Log Pseudo Marginal Likelihood (LPML) method [2] involves assessment using the Conditional Predictive Ordinate, which represents the predictive density of an observation in the estimated model without it. This approach selects models based on their predictive capacity, computing a statistic that indicates the optimal model to use. However, the obtained statistic does not enable us to determine whether the utilized model is a good fit; rather, it only indicates if it is superior to the others with which it was compared.

Gelman et al. [1] proposed another method based on external validation in which a predictive interval of probability 0.5 is computed for observations not utilized in the modeling process. This involves assessing the number of observations falling within these intervals, as it should closely align with the defined 50% credibility ([1], p. 142). Despite its intuitive nature, this approach is not widely adopted due to its subjective rejection criterion, which can vary based on the user’s perspective on quality assessment. In this context, this work aims to adapt the external validation methodology proposed by [1], as it provides an intuitive approach to assessing the accuracy of a model. To achieve this goal, adjustments will be made to certain steps of the method, facilitating the establishment of an objective criterion for evaluating the model’s quality.

## 2. Proposal for Analysis of Predictive Capacity

This study proposes an adaptation of the external validation approach suggested by [1] to evaluate a model’s quality through the predictive capacity of its posterior distribution. The use of the posterior distribution ensures the suitability of the final model, as methods that solely assess the likelihood function may not ensure the appropriateness of the prior distribution used, potentially compromising the final model’s outcomes. The proposed method entails employing the leave-one-out (LOO) technique to assess the model’s ability to accurately predict new observations. The procedure consists in calculating the proportion of correctly predicted values and uses it as a quality statistic. It checks whether the observed value is feasible given the chosen credible level and rejects the model when the observed proportion is unlikely. This idea is consistent with the emphasis on model prediction analysis that is common to both schools of thought of 20th century statistics (see [11] for more details) and can be efficiently implemented in a great variety of statistical models.

### 2.1. The Accuracy Measure

Let $C_{i}$ be a credible interval for the predicted value considering a fitted model without observation *i* from the sample, where i=1,2,…,n. If the value $y_{i}$ falls within the predicted interval, it is classified as a correct prediction ($u_{i}=1$); otherwise, it is classified as an error ($u_{i}=0$), i.e.,

(1) $$u_{i}=\left\{\begin{array}{c} 1,y_{i}\in C_{i}\\ 0,y_{i}\notin C_{i} \end{array}\right.,\;i=1,2,\ldots ,n.$$

Thus, the proportion of correct predictions is given by

(2) $$\kappa =\sum_{i=1}^{n}\frac{u_{i}}{n}.$$

The LOO technique prevents the double use of data, unlike the Posterior Predictive Check. Moreover, employing interval estimators simplifies specifying an expected proportion of accurate predictions for the model, as for a γ × 100% credible interval, this proportion should be close to γ, 0 < γ < 1. Consequently, a κ value far from γ suggests the model lacks good predictive capacity and is not suitable for representing the problem. Therefore, the proposed accuracy measure is determined by the difference between the proportion of accurate predictions and the credible level of the interval

(3) Δ=κ−γ.

The value of Δ ranges from −γ to 1−γ, and a value of Δ=0 indicates a good model accuracy. It is important to note that a proportion of correct predictions significantly higher than the credibility used (Δ>0) is not beneficial, as it indicates imprecision in the predictive interval. Alternatively, the more negative the value of Δ, the stronger the indication that the model has low predictive capacity. The proposed method shares a similar rationale with the Log Pseudo Marginal Likelihood (LPML), but it serves different objectives. Whereas the LPML is used for model comparisons, the aim here is to determine if a model can effectively predict a new observation.

### 2.2. Decision Criterion

We can construct a hypothesis test for the methodology, providing an objective approach to determine whether there is evidence that the model used lacks good predictive capability for the given problem. Consider the following hypotheses:

(4) $$\left\{\begin{array}{c} \;H:\;\mathrm{The}\;\mathrm{model}\;\mathrm{has}\;\mathrm{good}\;\mathrm{predictive}\;\mathrm{capability}.\\ H_{a}:\;\mathrm{The}\;\mathrm{model}\;\mathrm{does}\;\mathrm{not}\;\mathrm{have}\;\mathrm{good}\;\mathrm{predictive}\;\mathrm{capability}. \end{array}\right.$$

Hypothesis (4) can be tested using Bayesian hypothesis testing (see, for example, [12,13]) to determine if the proportion of correct predictions, κ, is equal to the credibility γ (i.e., Δ=0). Thus, hypothesis (4) can be reformulated as:

(5) $$\left\{\begin{array}{c} H:\kappa =\gamma \\ H_{a}:\kappa \neq \gamma . \end{array}\right.$$

Assuming a prior distribution $\kappa ∼Beta(a_{1},a_{2})$ and $u_{i}|\kappa ∼Bernoulli\left(\kappa \right)$, we obtain the posterior distribution of the proportion of correct predictions, given the observations, as $\kappa |u∼Beta(A_{1},A_{2})$, where $A_{1}=a_{1}+\sum_{i=1}^{n}u_{i}$ and $A_{2}=a_{2}+n-\sum_{i=1}^{n}u_{i}$. Here, $u_{i}$ (i=1,2,…,n) is given by Equation (1). Moreover, hypothesis (5) can be tested using the evidence value (*e*-value) of a Full Bayesian Significance Test (FBST) [12]. The *e*-value for testing hypothesis (5) can be obtained through Monte Carlo simulation following the steps of Algorithm 1.
**Algorithm 1:** Obtaining the *e*-value to test hypothesis (5).Generate $\kappa_{1},\kappa_{2},\ldots ,\kappa_{M}$ from a $Beta(A_{1},A_{2})$ distribution with parameters $A_{1}=a_{1}+\sum_{i=1}^{n}u_{i}$ and $A_{2}=a_{2}+n-\sum_{i=1}^{n}u_{i}$;Calculate the posterior density under *H*$:f^{H}\left(\gamma \right)=\frac{1}{B(A_{1},A_{2})}\gamma^{A_{1}-1}{\left(1-\gamma \right)}^{A_{2}-1}$;Calculate the posterior density: $f\left(\kappa_{m}\right)=\frac{1}{B(A_{1},A_{2})}\kappa_{m}^{A_{1}-1}{\left(1-\kappa_{m}\right)}^{A_{2}-1},m=1,\ldots ,M$;If $f\left(\kappa_{m}\right)\leq f^{H}\left(\gamma \right)$, set $v_{m}=1,m=1,\ldots ,M$;Calculate the *e*-value: $e-value=\frac{1}{M}\sum_{m=1}^{M}v_{m}$.

Note: In Steps 2 and 3, $B\left(A_{1},A_{2}\right)=\int_{0}^{1}z^{A_{1}-1}{\left(1-z\right)}^{A_{2}-1}dz$ is the beta function.

Below, we provide R code for obtaining the *e*-value to test hypothesis (5).


 *#* *Kappa*_*h* = *Kappa value under* *H* 
 *#* *M*       = *Number of Monte Carlo replicates*  
 *#* *a*, *b*    = *Prior hyperparameters*  
 *#* *n*       = *Sample size*  
 *#* *u*       = *Number of correct predictions* 
 Kappa     <- rbeta(M,a+u,b+n-u) 
 f_post    <- dbeta(Kappa,a+u,b+n-u) 
 f_post_h  <- dbeta(Kappa_h,a+u,b+n-u) 
 e.value   <- sum(f_post<=f_post_h)/M 
 e.value
		

In this work, we opted for the level γ=0.5 since it results in symmetry in the lower and upper deviations. Note that this symmetry does not hold for γ≠0.5. For γ=0.95, the situation where the proportion of correct predictions is less than the credible level (Δ<0) is less concerning than when the proportion of correct predictions is greater than the credible level (Δ>0).

According to the FBST, hypothesis *H* is rejected, meaning the proportion of correct predictions is different from 0.5 (or the model does not exhibit good predictive capability), if *e*-value < α. Here, α is the “critical value” fixed or obtained from elicited loss functions [14].

Alternatively, according to the methodology outlined in this work, we reject the null hypothesis *H* if $|\Delta_{obs}|\;>\Delta_{critical}$, where $\Delta_{critical}$ depends on the critical value α and the sample size *n*.

**Figure 1:**
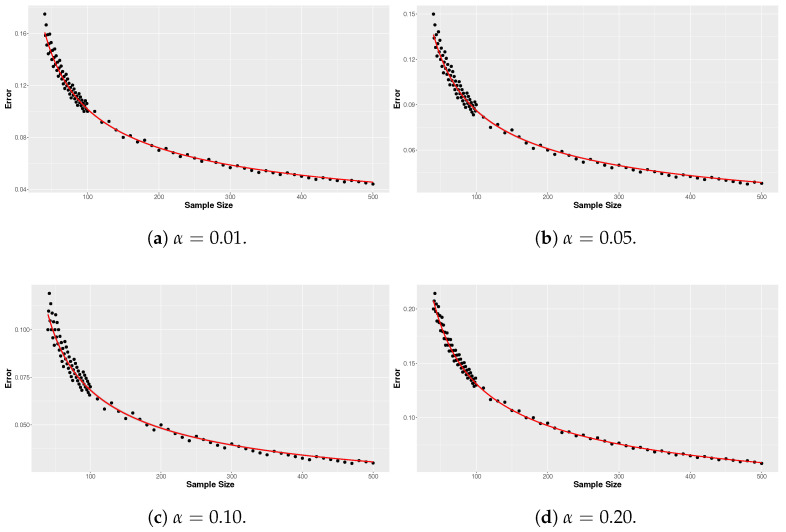
Regression curves of the error ξ.

To establish the critical points for the rejection criterion, samples ranging from *n* = 10 to 500 were generated. To determine the critical points for other values of *n*, a least squares regression was performed for the errors ξ=|Δ| using the square root of the sample size as the explanatory variable. Notice that the adopted value γ = 0.5 results in symmetry in the error ξ. This regression was adjusted to allow interpolation and extrapolation for n>40. The regression model adopted was $\xi =\frac{\beta_{1}}{\sqrt{n}}$. The estimated parameters for the regression curves with α = 0.01, 0.05, 0.1, and 0.2 were, respectively, $\beta_{1}$ = 1.261, $\beta_{1}$ = 0.966, $\beta_{1}$ = 0.812, and $\beta_{1}$ = 0.633. The values of $\Delta_{critical}$ for α = 0.01, 0.05, 0.1, and 0.2 were obtained from the FBST procedure considering the Beta(1,1) as prior distribution and *M* = 1,000,000 Monte Carlo replicates. Figure 1 displays the curve fits for the errors associated with sample size for different values of α. These graphs demonstrate satisfactory adjustments, indicating that the regression equations aptly represent the errors.

Table 1 presents the values of $\Delta_{critical}$ for *n* = 10 to 40 as well as its approximation for n>40.

## 3. Two Simple Examples

### 3.1. Exponential Distribution

Consider, for example, the exponential distribution, widely used in fields such as health and reliability. This distribution was chosen for its single parameter, which simplifies the comprehension of the proposed methodology. Let $X_{1},X_{2},\ldots ,X_{n}$ be a sample from *X*, which follows an exponential distribution with mean $\frac{1}{\theta }$, i.e., X|θ∼Exponential(θ). Assuming a priori θ∼Gamma(a,b),a,b>0, we obtain the posterior distribution θ|X∼Gamma(a+n,$b+\sum_{i=1}^{n}x_{i})$. Thus, the predictive density function of a new observation Y|X is given by:

(6) $$f_{Y|X}\left(y\right)=\left(a+n\right)\frac{{\left(b+\sum_{i=1}^{n}x_{i}\right)}^{a+n}}{{\left(y+b+\sum_{i=1}^{n}x_{i}\right)}^{a+n+1}},y>0.$$

Therefore, the quantile *q* of Y|X is

(7) $$y_{q}=\left(b+\sum_{i=1}^{n}x_{i}\right)\left[{\left(1-q\right)}^{\frac{-1}{a+n}}-1\right],$$

resulting in the following equal-tailed γ × 100% credible interval for the predicted value *y*, given the sample $X_{1},X_{2},\ldots ,X_{n}$:

(8) $$CI_{\gamma \times 100\%}:\left\{\left(b+\sum_{i=1}^{n}x_{i}\right)\left[{\left(1-\frac{\gamma }{2}\right)}^{\frac{-1}{a+n}}-1\right];\left(b+\sum_{i=1}^{n}x_{i}\right)\left[{\left(\frac{\gamma }{2}\right)}^{\frac{-1}{a+n}}-1\right]\right\}.$$

The percentage of correct predictions and the proposed accuracy measure in this work can be obtained through the steps outlined in Algorithm 2.
**Algorithm 2:** Obtaining the accuracy measure Δ.Set i=1;Create sample $S_{i}$ by removing observation *i* from the complete dataset;From $S_{i}$, obtain the credible interval $C_{i}$ for a new observation;Check if the observation *i*, removed from the sample, lies within the predicted interval:
(a)If the observation *i* lies within the credible interval, set $u_{i}=1$;(b)If the observation *i* does not lie within the credible interval, set $u_{i}=0$;If i<n, set i=i+1 and return to Step 2;Calculate the proportion of correct predictions, κ, using Equation (2);Calculate the accuracy measure, Δ, using Equation (3).

In situations where obtaining the predictive distribution is not feasible, it can be numerically estimated using MCMC—Markov Chain Monte Carlo [15]. To obtain a numerical approximation of the credible interval mentioned in Step 3, the following procedure can be used:

- For j=1,…,J, generate $\theta^{\left[j\right]}$ from the posterior distribution of θ|X.
- For each value of $\theta^{\left[j\right]}$, generate $y_{i}^{\left[j\right]}∼Exponential\left(\theta^{\left[j\right]}\right)$. Thus, $y_{i}^{\left[1\right]},y_{i}^{\left[2\right]},\ldots ,y_{i}^{\left[J\right]}$ is a sample from the predictive distribution (6).
- The limits of the equal-tailed credible interval for a new observation $y_{i}$ are given by the quantiles $\frac{\gamma }{2}$ and (1 – $\frac{\gamma }{2}$) of $y_{i}^{\left[1\right]},y_{i}^{\left[2\right]},\ldots ,y_{i}^{\left[J\right]}$. Alternatively, the HPD (highest posterior density) interval can be obtained from $y_{i}^{\left[1\right]},y_{i}^{\left[2\right]},\ldots ,y_{i}^{\left[J\right]}$ by using the emp.hpd command from the TeachingDemos package in R [3].

A drawback of the proposed method is its high computational cost, as it requires estimating a model for each observation in the sample, which can be inefficient for large datasets. In such situations, ref. [10] presented an optimized computation method for LOO using Pareto-smoothed importance sampling. This method effectively manages importance weights and is conveniently implemented in the *loo* package within the R programming environment [3].

**Figure 2:**
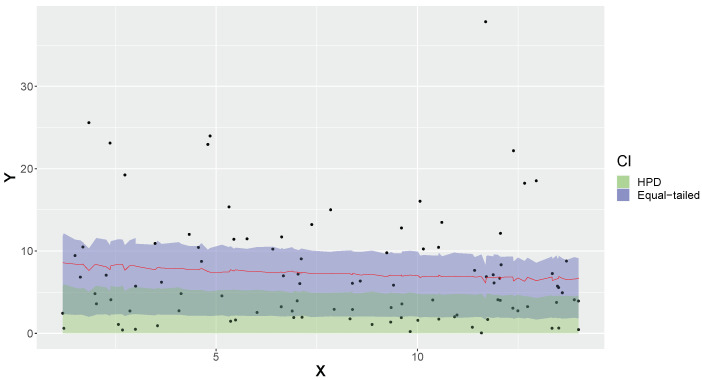
Observed values and 50% credible intervals of a new predicted observation in exponential model.

Figure 2 depicts data generated from a sample of size *n* = 100 from an exponential distribution and its predictive intervals (HPD and equal-tailed) with 50% credibility (γ=0.5) calculated from Equation (6). The analysis was performed considering a diffuse prior Gamma$\left(a=100^{-1},b=100^{-1}\right)$ for θ. In an asymmetric distribution, as is the case of Equation (6), the HPD and equal-tailed intervals will present distinct regions despite having the same credibility (Figure 2). The fact that the HPD interval does not contain the mean is due to the used credibility and the asymmetry of the predictive distribution, as the HPD interval is dependent on the mode rather than the mean of the distribution. In this example, the exponential distribution exhibited a good predictive fit, with the proportions of correct predictions being 47% and 53% for the 50% equal-tailed and HPD credible intervals, respectively. Note that both types of intervals resulted in 0.030 = $|\Delta_{obs}|$ < $\Delta_{critical}=\frac{0.966}{\sqrt{n}}$ = 0.097 (Table 1; *n* = 100; α = 0.05), which leads to non-rejection of the hypothesis that the exponential model has good capacity to predict future data. For observed accuracy rate κ = 0.47 (and κ = 0.53), the FBST yielded an *e*-value of 0.545, also leading to non-rejection of the hypothesis for α = 0.05.

### 3.2. Poisson Distribution

Let $X_{1},X_{2},\ldots ,X_{n}$ be a sample from *X*, which follows a Poisson distribution with mean θ, i.e., X|θ∼Poisson(θ). Assuming a priori θ∼Gamma(a,b),a,b>0, we obtain the posterior distribution $\theta |X∼Gamma(a+\sum_{i=1}^{n}x_{i},b+n)$. Thus, the predictive distribution of a new observation Y|X is given by a Gamma–Poisson distribution (with parameters $A=a+\sum_{i=1}^{n}x_{i}$ and B=b+n):

(9) $$f_{Y|X}\left(y\right)=\frac{\Gamma (a+\sum_{i=1}^{n}x_{i}+y)}{\Gamma \left(a+\sum_{i=1}^{n}x_{i}\right)\Gamma \left(y+1\right)}\frac{{\left(b+n\right)}^{a+\sum_{i=1}^{n}x_{i}}}{{\left(b+n+1\right)}^{a+\sum_{i=1}^{n}x_{i}+y}},y=0,1,\ldots .$$

Therefore, the lower and upper limits of the equal-tailed γ × 100% credible interval for the predicted value *y*, given the sample $X_{1},X_{2},\ldots ,X_{n}$ can, respectively, be obtained by $L_{1}=\sup\left\{y:F_{Y|X}\left(y\right)\leq \frac{\gamma }{2}\right\}$ and $L_{2}=\inf\left\{y:F_{Y|X}\left(y\right)\geq 1-\frac{\gamma }{2}.\right\}$, where $F_{Y|X}\left(y\right)$ is the cumulative predictive distribution given by $F_{Y|X}\left(y\right)=\sum_{k=0}^{y}f_{Y|X}\left(k\right)$.

**Figure 3:**
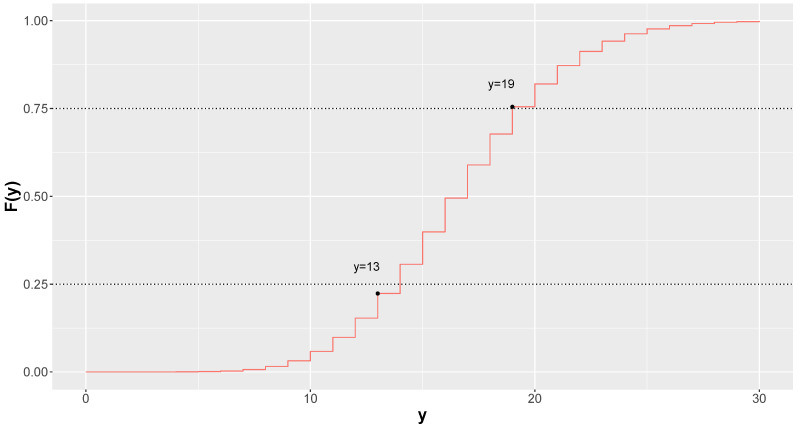
Cumulative distribution function of Gamma–Poisson distribution with lower and upper limits of the 50% credible interval. The real credibility of the interval is 60.2%.

As an example, consider that Y∼Gamma-Poisson(502,30). In this case, the limits of the equal-tailed 50% credible interval are given by $L_{1}$ = 13 and $L_{2}$ = 19. Figure 3 presents the cumulative distribution function of *Y*.

It is important to emphasize that since the predictive distribution is discrete, the interval may not have credibility (exactly) equal to γ. In fact, the real credibility of the interval will be greater than or equal to γ. Therefore, the test proposed in this paper will be approximate in cases where the predictive distribution is discrete. An alternative in these cases is to consider in hypothesis test (5) the average credibility of each of the *n* intervals obtained in the LOO steps.

**Figure 4:**
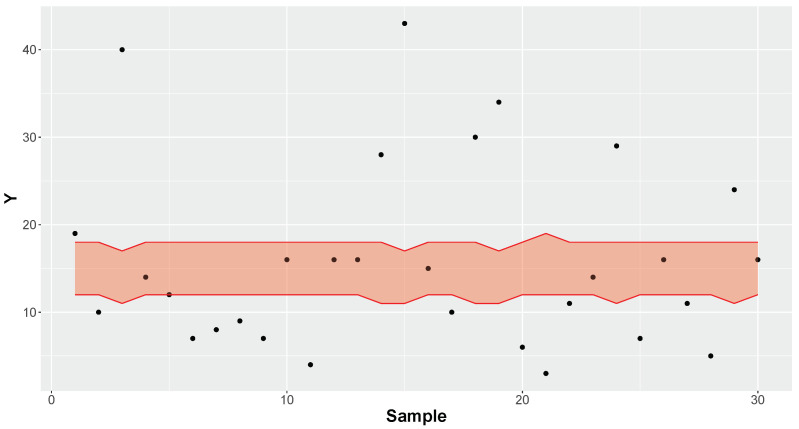
Observed values and 50% credible intervals (region in red) of a new predicted observation in Poisson model. The average credibility of the intervals is 63.2%.

Figure 4 depicts data generated from a sample of size *n* = 30 from a Negative Binomial distribution and its predictive equal-tailed intervals with 50% credibility estimated by a Poisson model. The analysis was performed considering a diffuse prior $Gamma(a=100^{-1},b=100^{-1})$ for θ. As expected, the Poisson model exhibited a poor predictive fit, with the proportion of correct predictions being 30% for the 50% equal-tailed credible intervals. This observed proportion of correct predictions resulted in 0.2 = $|\Delta_{obs}|$ > $\Delta_{critical}$ = 0.183 (Table 1; *n* = 30; α = 0.05), which leads to rejection of the hypothesis that the Poisson model has good capacity to predict future data. For observed accuracy rate κ = 0.3, the FBST yielded an *e*-value of 0.023, also leading to rejection of the Poisson model for α = 0.05. In addition, the FBST of hypothesis (5) considering $\kappa^{*}$ = 0.632 (the average credibility of each *n* = 30 intervals obtained in the LOO steps) yielded *e*-value < 0.001, also leading to rejection of the Poisson model for α = 0.05.

## 4. Simulation Study

**Figure 5:**
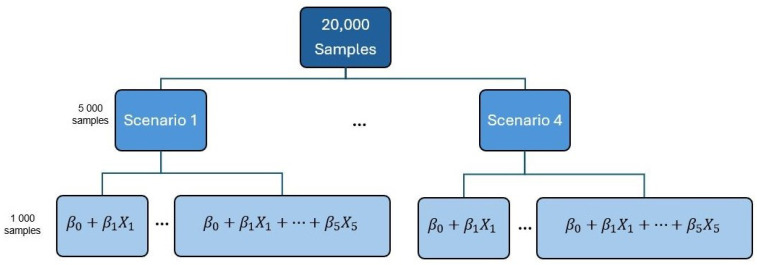
Simulation structure.

In this section, we present a simulation study to verify whether factors such as the nature of the covariates (numeric or categorical), the number of model parameters, or the sample size used for estimation could potentially influence the behavior of the proportion of correct predictions, κ, making it crucial to investigate their effects in determining the critical value. To assess which of these factors truly impact the value of κ, simulation studies were conducted using exponential regression models. To examine the effects of possible interactions among the factors, samples of size *n* were simulated, considering four scenarios of parameters with one to five predictors each, resulting in twenty distinct scenarios. For each of these scenarios, 1000 samples were generated, totaling 20,000 samples. The methodology was then applied to each of these generated samples. The simulated values of *n* ranged from 10 to 40, 50, 60, *…*, 140, and 150. Given the ease of obtaining and interpreting results, this study will solely use the equal-tailed interval to define the κ value. Therefore, simulations will be conducted exclusively with equal-tailed intervals. The flowchart depicted in Figure 5 illustrates the structure used in the simulation, along with the scenarios of parameters utilized.

The scenarios were chosen to maximize the diversity of parameter values and covariates used. Below are the four scenarios considered:

$$S_{1}:X^{T}\beta =-0.7+1.1X_{1}-0.6X_{2}+0.2X_{3}+X_{4}-1.5X_{5}$$

$$S_{2}:X^{T}\beta =1-1.3X_{1}+0.4X_{2}-0.2X_{3}+0.9X_{4}-0.3X_{5}$$

$$S_{3}:X^{T}\beta =-0.3+0.7X_{1}-1.2X_{2}+1.1X_{3}-0.7X_{4}+X_{5}$$

$$S_{4}:X^{T}\beta =1.7-0.8X_{1}+0.1X_{2}+0.6X_{3}-0.8X_{4}-1.1X_{5}$$

In these scenarios, $X_{1}∼Uniform\left(a=0,b=5\right)$, $X_{2}∼Bernoulli\left(p=0.2\right)$, $X_{3}∼Normal\left(\mu =0,\sigma^{2}=1\right)$, $X_{4}∼Bernoulli\left(p=0.7\right)$ and $X_{5}∼Uniform\left(a=0,b=5\right)$.

**Figure 6:**
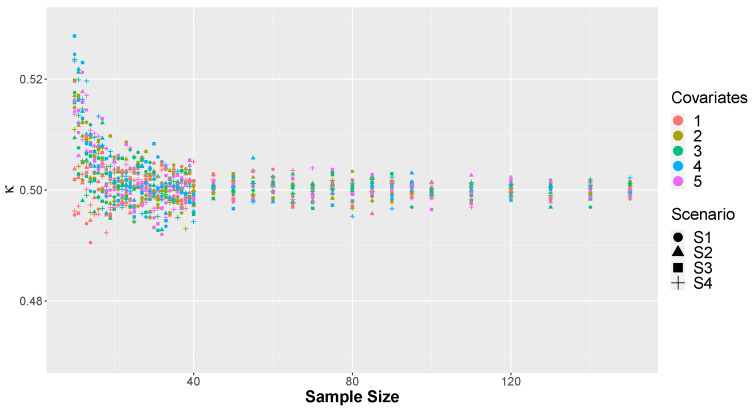
Average proportion of correct predictions, κ, by scenario and number of covariates.

**Figure 7:**
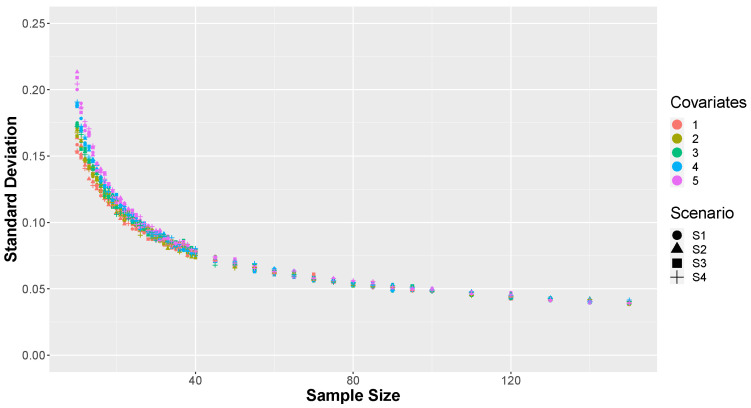
Standard deviation of the proportion of correct predictions, κ, by scenario and number of covariates.

In Figure 6 and Figure 7, we can observe the mean and standard deviation of the κ values for each of the *M* = 20,000 Monte Carlo replicates across the four considered scenarios of factors. It is noticeable that in small sample sizes, significant disparities were observed among models with varying numbers of covariates. Simulations with higher numbers of covariates exhibited higher means and deviations compared to others. This outcome is expected due to model saturation with small samples, attempting to estimate numerous parameters with limited observations, resulting in lower predictive capacity of the adjusted model. However, as the sample size increases, differences based on the number of covariates diminish, and all models converge to the same value in terms of both mean and standard deviation. It is evident that when the model has fewer covariates, approximately less than 20% of the sample size, the value of κ is not affected by this factor.

The various scenarios of parameters used did not affect the value of κ, as it showed well-distributed values across the simulations. This suggests that the types of covariates do not significantly influence the model’s accuracy percentage. Another expected outcome is the convergence of the standard deviation of the proportion of correct predictions to zero, with the mean converging to 0.5. This occurs because as the sample size increases, there is a greater concentration of correct predictions around the chosen credible level.

**Figure 8:**
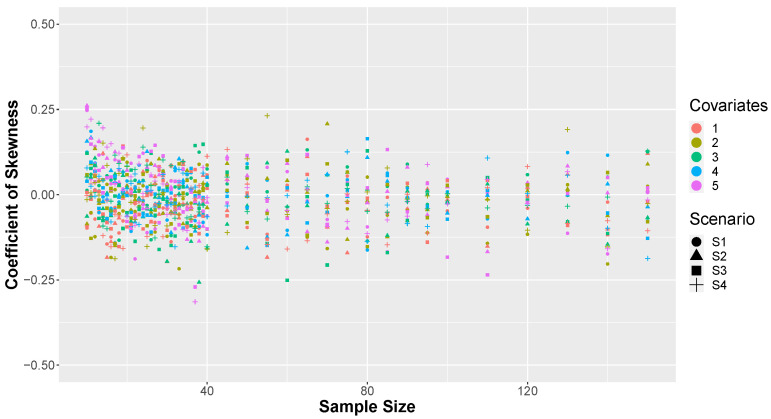
Skewness of κ by number of covariates.

To assess whether all simulated proportions of correct predictions exhibited symmetrical behavior, the skewness coefficient was calculated for the number of covariates, type of combination, and sample size. Figure 8 presents the results of the skewness coefficients calculated for the simulations. It can be observed that all values cluster close to 0, indicating evidence of symmetry in all simulated κ values. The slight fluctuations around zero are a result of the number of simulations conducted in each scenario. Nonetheless, values ranging from −0.25 to 0.25 are very close to symmetrical behavior and can be approximated without sacrificing accuracy.

Based on the results from the cross-simulations, it was determined that only the sample size factor has an impact on the proportion of correct predictions, κ. As a result, the sample size, *n*, will solely be used to establish the model rejection criterion.

**Figure 9:**
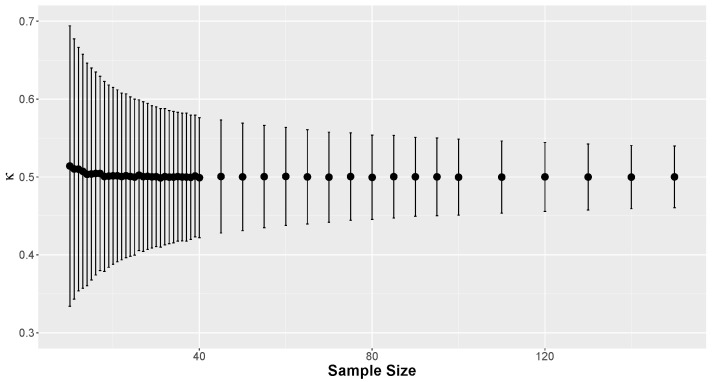
Average and standard deviation of κ by sample size.

Figure 9 displays the average and standard deviation of the simulated accuracy proportions, κ, based on the sample size. Each data point on the graph represents 20,000 simulations, enhancing the precision of the estimates.

**Figure 10:**
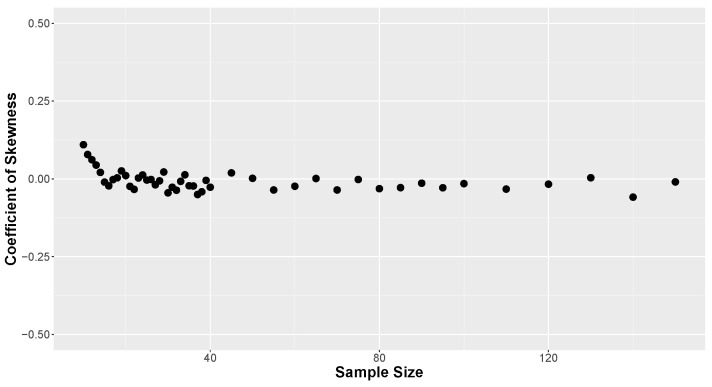
Skewness of κ by sample size.

The average values of κ are consistently centered around 0.5, reflecting the chosen credible level. Additionally, as the sample size increases, the standard deviation tends to zero, as seen in the previous results. Skewness was also calculated for the aggregated proportion of correct predictions based solely on the sample size, with the results displayed in Figure 10. These coefficients are very close to 0, indicating equal-tailed distributions.

The findings from this simulation study indicate that only the sample size factor needs to be considered in formulating the rejection criterion, showing that the critical points presented in Table 1 are valid regardless of the type and number of explanatory variables in the model.

## 5. Illustrative Example

The Leukemia dataset, as presented by [16], contains information on the time of death (in weeks) and the white blood count for two groups of leukemia patients, totaling 33 observations. The data are presented in Table 2.

In this application, the model proposed was an exponential regression with parameter $\theta =e^{-(\beta_{0}+\beta_{1}\times WBC+\beta_{2}\times AG)}$, where WBC represents the quantity of white blood cells (measured in units of 10,000) and AG denotes the presence of *Auer rods* and/or significant granulation of the leukemic cells in the bone marrow at the time of diagnosis (AG Present = 1 and AG Absent = 0). The proposed methodology was applied to the Leukemia dataset using a diffuse prior $N(\mu =0,\sigma^{2}=100^{2})$ for $\beta_{0}$, $\beta_{1}$, and $\beta_{2}$. This involved generating 1,000,000 samples with thinning interval of 5 and a burn-in of 10,000 in the MCMC process. The results from the LOO technique for assessing predictive capacity using this methodology are presented in Table 3 and Figure 9.

**Figure 11:**
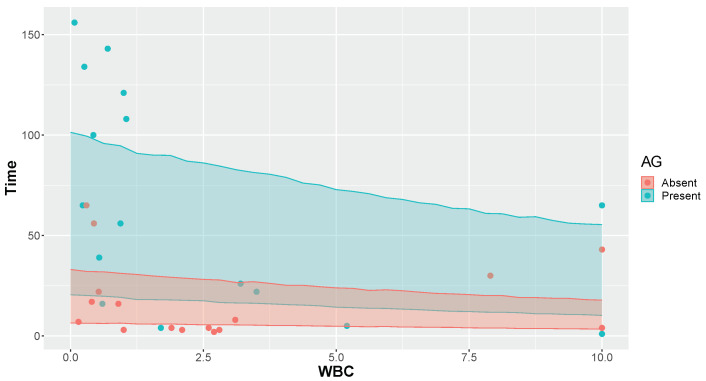
The 50% credible intervals obtained by the LOO technique.

From Figure 11, it is evident that the predictive capacity for individuals with AG Present was unsatisfactory, as a significant number of points lie outside the 50% credible interval. This indicates a potential poor fit of the model to the data. In this application, the observed accuracy rate was $\kappa =\frac{11}{33}=0.333$ (Table 3), resulting in $\Delta_{obs}=0.333-0.5=-0.167$. Referring to Table 1 for n=33, we find $\Delta_{critical}=0.152$ for α=0.05. Thus, with 95% credibility, we reject the hypothesis that the exponential model used has good predictive capacity for the problem, since $|\Delta_{obs}|\;>\Delta_{critical}$.

For observed accuracy rate κ = 0.333 (and *n* = 33), the FBST yielded an *e*-value of 0.048, also leading to the rejection of the hypothesis for α = 0.05. It is noteworthy that both decision criteria fell into regions that reject the hypothesis, demonstrating suitability in the decision criterion based on critical values for Δ presented in Table 1.

It is important to mention that the choice of prior distribution impacts directly on the posterior distribution and its misspecification can result in low model accuracy. As an example, consider changing the prior distribution of $\beta_{0}$ to an informative prior $N(\mu =0,\sigma^{2}=1)$ and keeping the same diffuse prior $N(\mu =0,\sigma^{2}=100^{2})$ for $\beta_{1}$ and $\beta_{2}$. With this change, we obtain an observed accuracy rate of $\kappa =\frac{10}{33}=0.303$, resulting in $\Delta_{obs}=-0.197$. This result indicates that the proposed accuracy measure identified the loss of accuracy due to misspecification of the prior distribution.

In addition, a measure usually used to assess the accuracy of a model is the Root Mean Squared Error (*RMSE*), defined by $RMSE=\sqrt{\frac{1}{n}\Sigma_{i=1}^{n}(Y_{i}-{\hat{Y}}_{i}{)}^{2}}$. Here, ${\hat{Y}}_{i}$ is the point estimate of the predicted value of individual *i*, i=1,2,…,n, defined as the mean of the predictive distribution. When considering the diffuse prior $N(\mu =0,\sigma^{2}=100^{2})$ for $\beta_{0}$, $\beta_{1}$, and $\beta_{2}$ (results in Table 3), we obtain RMSE = 40.290. However, when replacing the prior distribution of $\beta_{0}$ by a misspecified informative prior $N(\mu =0,\sigma^{2}=1)$, the value increased to RMSE = 122.303. When comparing the accuracy of the two models using *RMSE*, it is clear that the first model is more accurate. However, the RMSE fails to identify that even the “more accurate model” does not present a good predictive capacity for the data in Table 2, as shown by the accuracy measure proposed in this paper.

## 6. Discussion

This study presented an adaptation of a methodology based on external validation proposed by [1], which, despite its simplicity and intuitiveness, lacked an objective way to validate models. The adaptation enabled the definition of an accuracy measure following the model rejection criterion, providing an objective way to validate models. Previously, discrimination could vary depending on the researcher’s perspective. The development of this proposal was carried out from a Bayesian perspective of inference, elucidating the concepts used in its formulation and outlining the necessary steps for its application.

The decision criterion was defined from the FBST (Full Bayesian Significance Test) procedures. The conducted simulation study based on generalized linear models with an exponential distribution indicated that the proposed accuracy measure depends solely on the sample size.

The application of this methodology to real data allowed us to confirm its ease of use and its ability to identify when a model lacks good predictive capability. With its intuitive simplicity, ease of implementation, and low complexity, it is believed that the methodology proposed in this study will become an attractive alternative for model evaluation. This may encourage more research in this field, given the promising results obtained.

## Figures and Tables

**Table 1:** Critical values of Δ for γ = 0.5.

*n*	$\Delta_{\mathrm{critical}}$			
	**α = 0.01**	**α = 0.05**	**α = 0.10**	**α = 0.20**
10	0.350	0.250	0.250	0.150
11	0.364	0.273	0.273	0.182
12	0.375	0.292	0.208	0.208
13	0.308	0.231	0.231	0.154
14	0.321	0.250	0.179	0.179
15	0.333	0.267	0.200	0.133
16	0.281	0.219	0.219	0.156
17	0.294	0.235	0.176	0.176
18	0.306	0.194	0.194	0.139
19	0.263	0.211	0.158	0.158
20	0.275	0.225	0.175	0.125
21	0.286	0.190	0.190	0.143
22	0.250	0.205	0.159	0.114
23	0.261	0.217	0.174	0.130
24	0.229	0.188	0.146	0.146
25	0.240	0.200	0.160	0.120
26	0.250	0.173	0.173	0.135
27	0.222	0.185	0.148	0.111
28	0.232	0.196	0.161	0.125
29	0.241	0.172	0.138	0.103
30	0.217	0.183	0.150	0.117
31	0.226	0.161	0.129	0.097
32	0.234	0.172	0.141	0.109
33	0.212	0.152	0.152	0.121
34	0.221	0.162	0.132	0.103
35	0.200	0.171	0.143	0.114
36	0.208	0.153	0.125	0.097
37	0.216	0.162	0.135	0.108
38	0.197	0.145	0.118	0.092
39	0.205	0.154	0.128	0.103
40	0.188	0.163	0.138	0.088
Approx. for n>40	$\frac{1.261}{\sqrt{n}}$	$\frac{0.966}{\sqrt{n}}$	$\frac{0.812}{\sqrt{n}}$	$\frac{0.633}{\sqrt{n}}$

Decision criterion: Reject *H* if $|\Delta_{obs}|>\Delta_{critical}$.

**Table 2:** Leukemia data.

AG Present (1)		AG Absent (0)	
**WBC**	**Time**	**WBC**	**Time**
0.23	65	0.44	56
0.075	156	0.3	65
0.43	100	0.4	17
0.26	134	0.15	7
0.6	16	0.9	16
1.05	108	0.53	22
1	121	1	3
1.7	4	1.9	4
0.54	39	2.7	2
0.7	143	2.8	3
0.94	56	3.1	8
3.2	26	2.6	4
3.5	22	2.1	3
10	1	7.9	30
10	1	10	4
5.2	5	10	43
10	65		

Source: Hand et al. [16].

**Table 3:** Results of the leave-one-out (LOO) technique for the data in Table 2 (exponential regression model).

Patient	Y	AG	WBC	$\beta_{0}$	$\beta_{1}$	$\beta_{2}$	$\hat{Y}$	Lower 50% CI	Upper 50% CI	Y in Interval
1	65	1	0.230	3.162	1.117	−0.064	73.713	19.931	99.966	Yes
2	156	1	0.075	3.138	1.044	−0.058	67.763	18.175	91.789	No
3	100	1	0.430	3.155	1.087	−0.063	70.059	19.012	94.907	No
4	134	1	0.260	3.145	1.059	−0.060	68.618	18.458	92.888	No
5	16	1	0.600	3.170	1.155	−0.066	75.156	20.362	102.077	No
6	108	1	1.050	3.156	1.074	−0.063	66.711	18.200	90.528	No
7	121	1	1.000	3.153	1.063	−0.062	65.988	17.922	89.522	No
8	4	1	1.700	3.161	1.171	−0.064	70.458	19.191	95.688	No
9	39	1	0.540	3.167	1.137	−0.066	73.575	20.010	99.818	Yes
10	143	1	0.700	3.147	1.046	−0.061	65.681	17.699	89.356	No
11	56	1	0.940	3.162	1.123	−0.064	70.542	19.162	95.804	Yes
12	26	1	3.200	3.154	1.152	−0.062	62.686	16.996	85.112	Yes
13	22	1	3.500	3.150	1.158	−0.062	62.087	16.709	84.311	Yes
14	1	1	10.000	3.087	1.214	−0.044	53.982	12.596	68.882	No
15	1	1	10.000	3.087	1.214	−0.044	54.119	12.587	68.914	No
16	5	1	5.200	3.131	1.185	−0.057	58.813	15.400	79.113	No
17	65	1	10.000	3.286	0.952	−0.091	33.086	7.168	41.304	No
18	56	0	0.440	2.983	1.263	−0.049	20.481	5.357	27.418	No
19	65	0	0.300	2.926	1.311	−0.043	19.621	5.124	26.274	No
20	17	0	0.400	3.187	1.090	−0.066	24.806	6.495	33.090	Yes
21	7	0	0.150	3.235	1.050	−0.071	26.568	6.941	35.657	Yes
22	16	0	0.900	3.189	1.089	−0.066	24.108	6.365	32.389	Yes
23	22	0	0.530	3.163	1.111	−0.064	24.115	6.327	32.342	Yes
24	3	0	1.000	3.244	1.040	−0.070	25.059	6.650	33.612	No
25	4	0	1.900	3.232	1.049	−0.068	23.231	6.242	31.377	No
26	2	0	2.700	3.233	1.045	−0.067	21.990	5.899	29.818	No
27	3	0	2.800	3.228	1.050	−0.067	21.785	5.863	29.525	No
28	8	0	3.100	3.206	1.069	−0.066	20.899	5.650	28.400	Yes
29	4	0	2.600	3.226	1.052	−0.067	21.959	5.923	29.687	No
30	3	0	2.100	3.235	1.045	−0.068	22.961	6.162	31.028	No
31	30	0	7.900	3.120	1.176	−0.076	13.203	3.442	17.634	No
32	4	0	10.000	3.167	1.088	−0.054	15.160	3.758	19.809	Yes
33	43	0	10.000	3.112	1.286	−0.122	7.357	1.768	9.539	No
			Full model	3.161	1.112	−0.064	RMSE = 40.290			

## Data Availability

The data presented in this study are from [16] and are also included in the article.

## Abbreviations

The following abbreviations are used in this manuscript:

CI	Credible Interval
FBST	Full Bayesian Significance Test
HPD	Highest Posterior Density
LOO	Leave-One-Out
LPML	Log Pseudo Marginal Likelihood
MCMC	Markov Chain Monte Carlo
RMSE	Root Mean Squared Error
WAIC	Widely Applicable Information Criterion
